# Chemical and Biological Molecules Involved in Differentiation, Maturation, and Survival of Dopaminergic Neurons in Health and Parkinson’s Disease: Physiological Aspects and Clinical Implications

**DOI:** 10.3390/biomedicines9070754

**Published:** 2021-06-29

**Authors:** Giulia Gaggi, Andrea Di Credico, Pascal Izzicupo, Giovanni Iannetti, Angela Di Baldassarre, Barbara Ghinassi

**Affiliations:** 1 Beth Israel Deaconess Medical Center, Harvard Medical School Initiative for RNA Medicine, Harvard Medical School, Boston, MA 02115, USA; ggaggi@bidmc.harvard.edu; 2 Human Anatomy and Cell Differentiation Lab, Department of Medicine and Aging Sciences, University “G. D’Annunzio” of Chieti-Pescara, 66100 Chieti, Italy; andrea.dicredico@unich.it (A.D.C.); izzicupo@unich.it (P.I.); b.ghinassi@unich.it (B.G.); 3 University of Rome La Sapienza, 00185 Rome, Italy; iannetti.1752004@studenti.uniroma1.it

**Keywords:** dopaminergic neurons, Parkinson’s disease, dopamine, miRNAs, lncRNAs, chemical small molecules, extracellular vesicles, exosomes, dopaminergic differentiation, stem cell differentiation

## Abstract

Parkinson’s disease (PD) is one of the most common neurodegenerative disease characterized by a specific and progressive loss of dopaminergic (DA) neurons and dopamine, causing motor dysfunctions and impaired movements. Unfortunately, available therapies can partially treat the motor symptoms, but they have no effect on non-motor features. In addition, the therapeutic effect reduces gradually, and the prolonged use of drugs leads to a significative increase in the number of adverse events. For these reasons, an alternative approach that allows the replacement or the improved survival of DA neurons is very appealing for the treatment of PD patients and recently the first human clinical trials for DA neurons replacement have been set up. Here, we review the role of chemical and biological molecules that are involved in the development, survival and differentiation of DA neurons. In particular, we review the chemical small molecules used to differentiate different type of stem cells into DA neurons with high efficiency; the role of microRNAs and long non-coding RNAs both in DA neurons development/survival as far as in the pathogenesis of PD; and, finally, we dissect the potential role of exosomes carrying biological molecules as treatment of PD.

## 1. Introduction

Parkinson’s disease (PD) is one of the most common neurodegenerative disorders, with an increasing incidence worldwide and a great effort for the health care resources [1,2]. At cellular level, PD is characterized by an irreversible and progressive loss of midbrain dopaminergic (DA) neurons in the substantia nigra pars compacta: this degeneration leads to the dysregulation of the nigrostriatal pathway that causes the manifestation of the clinical motor symptoms associated with PD [3,4].

Current therapeutic options for PD aim to support the nigrostriatal pathway administrating drugs that are able either to modulate the DA transmission or increase the dopamine level in the brain [3]. Unfortunately, these pharmacological treatments are only able to alleviate the physical symptoms, sometime delaying the disease progression [5], and their efficacy gradually reduces over the time [6]. For these reasons, an alternative approach with the aim to replace the degenerated cells with stem cell-derived DA neurons is appealing. In this scenario, human stem cell-derived models are particularly attractive because, unlike animal models, they avoid species-specific differences and can be patient-specific [7].

Based on their origin, stem cells can be classified into four categories: Embryonic, induced pluripotent, perinatal, and adult stem cells. Embryonic stem cells (ESCs), derived from a blastocyst, are pluripotent, being able to differentiate into the three germ layers, but their extended culture time in vitro results in chromosomal abnormality and instability [8]. Induced pluripotent stem cells (iPSCs) were obtained reprogramming adult cells using the ectopic expression of four transcription factors (OCT-4, SOX-2, Klf4, and c-myc) [7]. Perinatal stem cells can be isolated from the amniotic fluid, placenta, and umbilical cord. These cells cannot divide indefinitely in vitro; however, they are generally considered multipotent [9,10], but their real position in the stemness hierarchy is still unclear [11]. Adult stem cells reside within organs during post-natal life; they usually are oligo- or unipotent [12].

To date, functional human DA neurons can be generated with high efficiency only from ESCs or iPSCs using a multistep process that exploits a combination of chemical small molecules and growth factors; however, it has been recently highlighted that also biological molecules, such as extracellular vesicles (EVs), and non-coding RNAs can be involved in the protection or degeneration of DA neurons, leading to an improvement or a worsening of PD symptoms. This review provides an overview about the biological or synthetic molecules, involved in DA neuron development, that can favor the differentiation of stem cells into the DA fate and have a role in the prevention or in the treatment of PD.

## 2. Development of Midbrain DA Neurons

**Figure 1:**
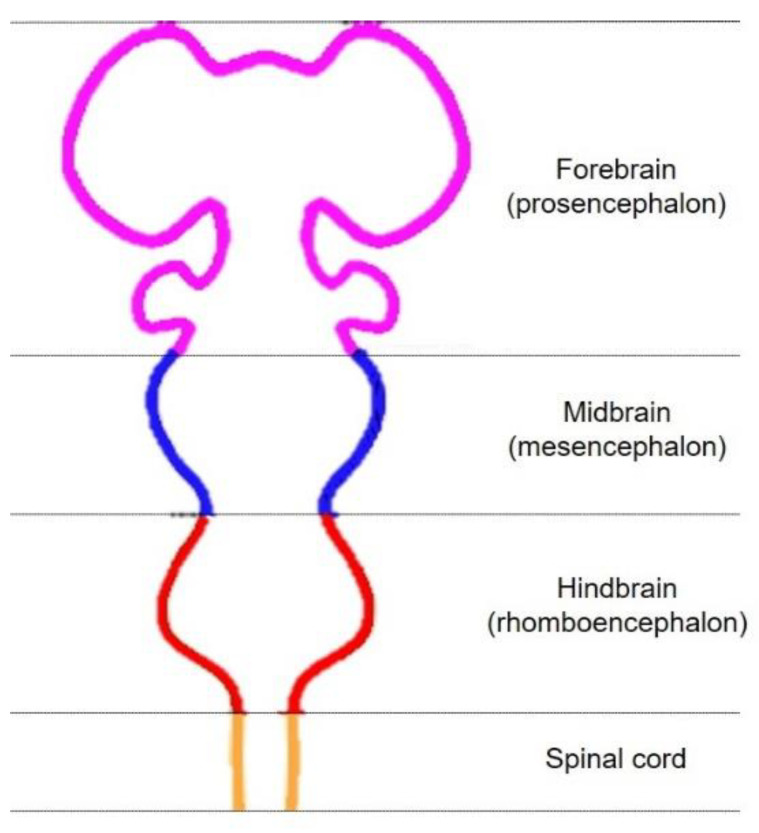
Schematic representation of developing neural tube.

During the early stages of mammalian embryonic development, the ectoderm becomes specified to give rise to the neural plate; this folds outward and creates the neural tube, which is initially divided into four parts: forebrain (prosencephalon), midbrain (mesencephalon), hindbrain (rhomboencephalon), and spinal cord [13] (Figure 1).

**Figure 2:**
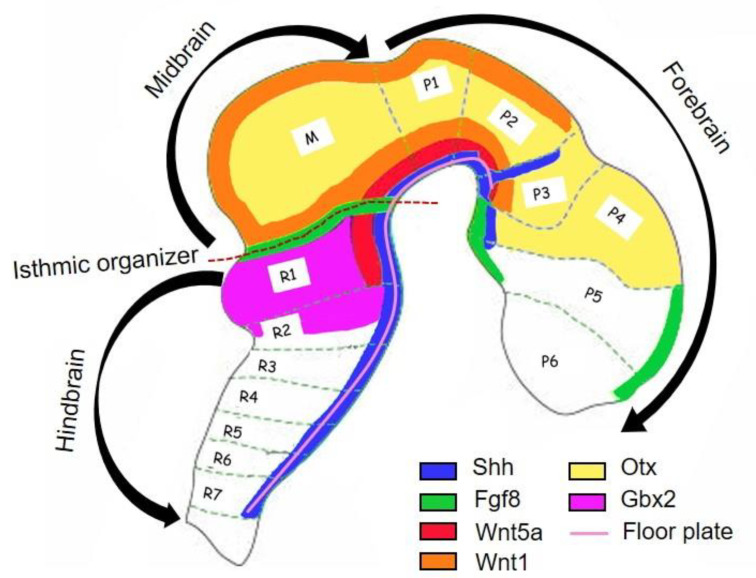
Schematic representation of signaling and morphogen involved in the formation of DA neurons. Otx and Gbx2 are two transcription factors acting antagonistically to set up the position of the IsO, that defines the midbrain–hindbrain boundary. IsO produces Fgf8, that together with Shh defines the region of midbrain DAminergic neurons development. Wnt1 and Wnt5 are expressed in this region and essential for the formation of midbrain (M). Prosomeres, P1–P6; Rhombmeres R1-R7.

As the development of the neural tube proceeds, two signaling centers form: the isthmic organizer (IsO), which defines the midbrain-hindbrain boundary, and the floor plate [14]. The correct positioning of IsO is due to the coordinated expression of the two transcription factors Otx2 and Gbx2 in the developing anterior neural tube: the first is expressed in the forebrain and midbrain, while Gbx2 in the anterior hindbrain [15]. Otx2 expression is limited caudally by Gbx and this limit corresponds to the border between midbrain and hindbrain [16] (Figure 2). As Otx2- and Gbx2-dependent sharpening of the IsO borders occurs, a second group of transcription factors is expressed by IsO, including Paired box gene 2 (Pax2), Lim homeobox transcription factor 1 beta (Lmx1b), and the secreted Wnt1 and Engrailed-1 (En1). In particular, Pax2 is required for the Fibroblast growth factor 8 (Fgf8) production by IsO, while Wnt1 and En1 cooperate with Otx2 and Gbx2 to further refine the expression domain of Fgf8 at the IsO [16,17]. These steps, together with the production of Sonic Hedgehog (Shh) by the floor plate, the other signaling center, are fundamental for the differentiation of progenitor cells into DA progenitor cells [3]: indeed, an orthogonal gradient of Shh and Fgf8 create a cartesian coordinate system that define the positional information for the midbrain DA phenotype induction (Figure 2). In addition, Shh activates the Glioma-associated oncogene 1 (Gli1), that is considered an early marker of midbrain DA progenitors [17].

**Figure 3:**
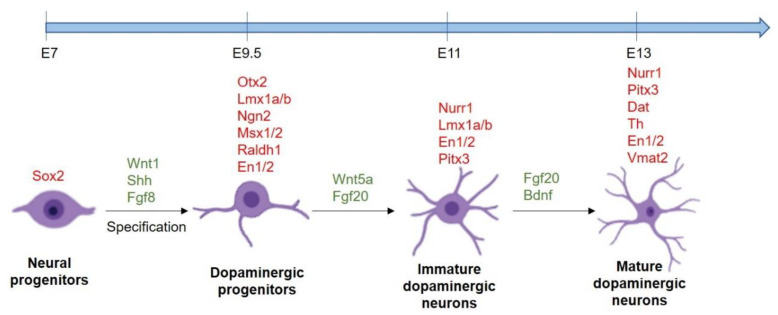
The timing of expression of transcription factors during the development of midbrain DA neurons. Transcription factors are indicated in red, whereas the main signaling pathways activated are indicated in green.

The first step in the development of midbrain DA neurons is the specification of floor plate cells into neural progenitor cells expressing Forkhead box protein A2 (Foxa2) and LIM homeobox transcription factor 1 alpha (Lmx1a): however, the factors and pathways controlling this transition are currently unknown [18,19]. These Foxa2^+^ and Lmx1a^+^ progenitor cells that reside in the ventricular zone of floor, initially proliferate, but subsequently they exit from the cell cycle and undergo a midbrain commitment: Lmx1a, indeed, activates the expression of Msh homeobox 1 (Msx1), that in turn induces the expression of Neurogenin 2 (Ngn2), thus generating post-mitotic midbrain DA progenitors that migrate to their final destination [3]. The importance of this passage is confirmed by the observation that Lmx1a and Ngn2 ablation leads to an absence or reduction in midbrain DA neuron number. During the migration, the midbrain DA progenitors continue to differentiate thanks to the concerted activation of transcription factors and Wnt and Shh signaling pathways [17]. Indeed, among the genes activated in the midbrain DA progenitors, Lmx1a together with Limx1b directly upregulate Nuclear receptor related-1 protein (Nurr1), an orphan nuclear receptor involved in the development of post-mitotic midbrain DA progenitors; then, Lmx1b and Nurr1 upregulate the expression of Pituitary homeobox 3 (Pitx3), a marker of mature DA neurons. Moreover, Nurr1 cooperates with Pitx3 for the expression of Thyroxine hydroxylase (Th), an enzyme that catalyzes the hydroxylation of tyrosine into L-Dopa, the precursor of DA. During the migration process the cells acquire also the expression of vesicular monoamine transporter (Vmat2), and the DA transporter (Dat), that are, respectively, involved in the transportation of DA into synaptic vesicles and its reuptake from the synaptic cleft [20] (Figure 3).

Recently it has been suggested that, in addition to the classical signaling pathways involved into the DA neurons development, also non-coding RNAs and exosomes carrying biological molecules may have an important role in the generation of DA neurons and that their deregulation can lead to an altered background causing the development of neurodegenerative diseases, such as PD, in the postnatal life [21,22].

## 3. Generation of DA Neurons from Stem Cells

In the last decade, the scientific community has deeply investigated the possibility to generate functional DA neurons differentiating stem cells. Despite the fact that human ESCs give rise to DA neurons with a high efficiency, their use hampers ethical issues [23]. iPSCs have been proposed as alternative to ESCs in 2006 [24]. Since their discovery, several groups have improved the reprogramming method to generate iPSCs from various somatic cells, including peripheral blood mononuclear cells [25] or keratinocytes [26]. One of the most advantage of the iPSCs technology resides in the possibility to generate them from the patient, avoiding the allograft rejection [27]. These advantages of iPSCs make the personalized and generalized cell therapy conceivable for further clinical application. On the other side, this technology needs further improvements to avoid the actual safety limitations due their tumorigenic potential together with the still too big differentiation efficiency that occur among the labs, probably due to the epigenetic memory of these cells [28]. For these reasons, an alternative stem cell source for the generation of DA neurons is still appealing for the scientific community.

The discovery of the existence of human adult neural stem cells in the hippocampus or subventricular zone of the lateral ventricles prompted scientists to think that these cells could have been the perfect candidate for neuronal regeneration [29], but their deep localization together with the rapid loss of differentiation capacity in in vitro culture [18] and low percentage of graft survival [30] limited their clinical application, making them a less ideal candidate for transplantation [31].

Recently, it has been demonstrated that functional DA neurons can be obtained also from human mesenchymal stromal cells (hFM-MSCs), a stem cell population that can be easily isolated from amniochorionic membrane after delivery and that, like all perinatal stem cells, their use does not have safety or ethical limitations [20], suggesting them as a candidate for regenerative medicine.

## 4. Chemical Compounds for Generation of DA Neurons from Stem Cells

Initially midbrain DA neurons were generated from human (h) pluripotent stem cells adapting protocols from mouse (m) ESCs, which generates neuronal rosette-like intermediate when co-cultured with feeder such as PA6 or M5 cell lines. Applying these neuronal-rosette-based protocols, DA neurons are generated expressing Th and releasing dopamine in vitro; however, these cells displayed a high risk of neural overgrowth in vivo [32]. After the discover that midbrain DA neurons originate from midbrain floor plate, a new class of differentiation protocol was then developed. They were based on the dual-SMAD inhibition and on the activation of SHH, WNT, and FGF8 pathways. The midbrain DA neurons obtained from this protocol showed a more robust functionality and survival in vitro, with a reduced risk of neural overgrowth and teratoma formation [32].

With the dual-SMAD inhibition, stem cells rapidly differentiate into early neurectoderm [33]. This rapid differentiation is caused by the block of the two signaling pathways that utilize SMADs for transduction: BMP and TGF-β. Starting from this dual SMAD inhibition, it is possible to generate DA neurons from hESCs, hiPSCs, and adult stem cells and, as recently evidenced, also from perinatal stem cells [20,34,35,36,37,38,39,40].

Several combinations of chemical compounds have been applied to obtain functionally differentiated DA neurons from different type of stem cells. Approximately 28 compounds were reported to be useful in inducing DA differentiation, when administered in combinations a specific timeline manner (see Table 1): SB431542, LDN193189, dorsomorphin, noggin, SHH, smoothened agonist, purmorphamine (PUR), CHIR99021, glial cell-derived neurotrophic factor (GDNF), brain-derived neurotrophic factor (BDNF), transforming growth factor beta-3 (TGF-β), ascorbic acid (AA), cyclic adenosine monophosphate (cAMP), N-[N-(3,-5-difluorophenacetyl)-L-alanyl]-S-phenylglycine t-butyl ester, interleukin 1β (IL-1β), basic FGF (also known as FGF2), FGF8, fibroblast growth factor 20, epidermal growth factor, laminin, heparin, WNT family member 1(WNT1), the γ-secretase inhibitor DAPT, retinoic acid (RA), leukemia inhibitory factor (LIF), endothelial growth factor D (EGF D), secreted frizzled related protein 1 (SFRP1), and stromal cell-derived factor 1a (SDF1) [41]. In particular, it has been shown that the early exposure to high level of SHH together with the two SMAD inhibition increased the expression of the FOXA2 marker, that is fundamental for the generation for the generation of DA progenitors [31].

Among the published protocols, one of the most cited is the one described by Kriks et al. [35], by which the authors were able to differentiate hESCs into DA neurons, that engrafted and survived for a long term in a mouse model, without any signs of neuronal overgrowth [35]. In comparison to previous protocols, the methods provided by Kriks et al. displayed the important characteristic of mimicking the steps of DA differentiation process in vivo. Indeed, first floor plate cells were generated both by the dual inhibition of SMAD signaling (by SB431542 and LDN193189) and the heavy activation of SHH pathway (by PUR and SHH administration); successively, the activation of WNT signaling induced the commitment into midbrain floor plate cells and finally the terminal differentiation was obtained treating cells with trophic factors, such as GDNF, BDNF, cAMP, TGF-β3, AA and DAPT, the latter with the aim to indirectly block the Notch signaling [35].

Starting from this protocol, several groups have then adapted the method to their cell lines and hundreds of papers have been published describing the production of DA neuros from hiPSCs and ESC by means of this multistep differentiation protocol. For example, Kirkeby et al. demonstrated that the modulation of Wnt signaling by different concentration of CHIR99021 can pattern the neural progenitors from the anterior to the posterior region, in particular a lower concentration of CHIR99021 drove the hESCs to the midbrain fate, whereas higher concentrations resulted in the caudal hindbrain fate [42]. On the other side, Takahashi’s group was the first team that introduced the sorting of the CORIN^+^ cells at 12 days of differentiation obtaining a very high percentage of FOXA2^+^/LMX1A^+^ DA neurons that were transplanted into both mouse and monkey models [36,43]. Another cell sorting protocol was developed by Lehnen et al., in which the integrin was used associated to protein (IAP), as a suitable cell marker for the enrichment of DA progenitor after the dual SMAD inhibition, obtaining an increase of the yield of mature DA neuros [37]. However, the percentage of TH^+^ DA neurons obtained using the dual-SMAD inhibition protocols is largely variable, with a differentiation efficacy that ranges from 8 to 85% among labs, and this might due more to the differences in hiPSCs clones and handling, rather than to the differentiation protocols [7]. Recently, it has been shown that also human mesenchymal stromal cells (hFM-MSCs) isolated from the amniochorionic membrane [44] can acquire a DA phenotype when treated with the small molecules cocktail used by Kriks et al. [20]. In the field of adult stem cells, valproic acid was shown to cooperate in DA differentiation of human spermatogonial stem cells when added to a conditioned medium from olfactory ectomesenchymal cells together with the small molecules SHH, RA, SB431542, FGF8, TGF-β3, and GDNF: the derived DA neurons cells were able to engraft and survive in a PD mouse model, partially rescuing the PD phenotype [34].

Table 1 shows an overview of some selected dual SMAD inhibition-based protocols for differentiation into midbrain DA neurons.

## 5. Chemical Compound for Generation of Human Midbrain Specific Organoids

Despite the development of monolayer protocols for the generation of DA neurons, which led to great progress in the study of the PD molecular mechanism, these classical two-dimensional (2D) cell culture models have some limitations. It has been demonstrated that they are not very accurate in recapitulating the spatial organization of neural tissues, cell–cell and cell–extracellular matrix interactions. For these reasons, the need for a more accurate models pushed towards the development of organoids, self-organized three-dimensional (3D) tissue cultures that are derived from stem cells [45]. Such cultures can be crafted to replicate much of the complexity of the embryonic brain, with wich share some gene expression features that are not recapitulated by 2D culture [46].

To date, many different approaches were published by different groups to generate 3D organoids displaying features resembling the ones of the human midbrain [46]. Tieng et al. were the first to generate human midbrain specific organoids (hMOs) adapting the 2D protocol published from Kriks et al. [35]. They created a 3D suspension using microwells that allow the generation of embryoid body with the same size that subsequently were grown on a semi-permeable PTFE membrane at the air–liquid interface, generating a population with 75% of TH^+^ cells [47]. A few years later, Qian et al. also developed a miniaturized spinning bioreactor that allowed the raising of TH^+^ and DAT^+^ hMO that recapitulated key features of human cortical development, including progenitor zone organization, neurogenesis, and gene expression, together with outer radial glia cell layer [48]. On the other side, Jo et al. [49], adapting the protocol of Chambers et al. [33], obtained DA neurons obtained electrically active inside hMO able to spontaneously produce neuromelanin-like granules similar to those accumulating in the substantia nigra pars compacta [49]. Monzel et al. described hMO, derived from neuroepithelial stem cells (hNESCs), that contained spatially organized groups of DA neurons with synaptic connections and electrophysiological activity other than the myelination of neurites [50]. Most recent evolutions of the 3D differentiation protocols aimed to establishing the best conditions to maximize the yield of DA neurons into the hMOs and making the hMO technology more reproducible. Recently, Kwak et al. developed a new differentiation protocol in which they combine the dual SMAD inhibition and the generation of a WNT gradient by specific concentration of CHIR99021 that increased the yield of TH^+^ neurons up to the 86% and efficiently reduced the cortical marker expressions. In addition, after 4 months of culturing, their hMO were able to produce high concentration of DA [51]. Table 2 summarized the most relevant protocol for the generation of hMOs. 

### Human Midbrain Organoids as Model of PD

In the context of PD, hMOs generated high expectations in the scientific community, as their 3D structures, recapitulating the architecture and the physiology of the human midbrain, may represent an in vitro model to understand the neurodevelopment and pathogenesis of PD [52,53].

It has been demonstrated that genetically modified or patient-derived hMOs carrying the G2019S mutation in the LRRK2 gene recapitulated the typical PD phenotype observed in patients with LRRK2-associated sporadic PD, including a reduced number of DA neurons and a lower number of branching, dendrite bifurcations, neurite length, suggesting that there was a significant reduction of the DA neurons network complexity in comparison with the control condition [52,54]. The LRRK2-G2019S gene correction within a PD patient background, anyway, was not sufficient to rescue the phenotype to the healthy condition, suggesting that other mechanisms can be implicated in the development of the disease [54]. In addition, many groups found a downregulation of DA markers, such as TH, VMAT2, DAT, and PITX3 both in hMO obtained from idiopathic and LRRK2-associated PD: this observation suggests that at the basis of the PD there is an alteration in the DA neuron development process [52,55]. The expression of some of these genes was partially recovered after the treatment of the mutant LRRK2-G2019S hMOs with a LRRK2 kinase inhibitor, suggesting that these 3D cellular model can be also used for drug screening and for the investigation of PD therapeutic options [52].

In the future, by deriving hMOs that carry genetic defects associated with the development of PD, scientists could broad the knowledge about the disease-related abnormalities in the midbrain. In addition, the generation of patient specific models paves the way for a future personalized medicine [46].

## 6. Biological Molecules Involved in Development of DA Neurons in Healthy and PD Condition

### 6.1. Biogenesis and Activity of microRNAs and Long Noncoding RNA

Recently, non-coding RNA-mediated post-transcriptional regulation of gene expression has emerged as a pivotal regulatory mechanism in neuronal differentiation [17]. These molecules exert a fine-tuning regulation of transcription factors and proteins involved in DA neuron development, dopamine signaling, and in the maintenance of functional and healthy DA neurons. This regulation is largely mediated by two groups of non-coding RNAs: microRNAs (miRNAs) and long noncoding RNAs (lncRNAs) [56]. miRNA genes are usually located in both intronic and exonic regions of protein-coding genes or in the intergenic regions of genome [57]. Generally, they are transcribed by RNA polymerase II or III into primary miRNA transcripts (pri-miRNA), which range from hundreds to thousands of nucleotides in length.

Within the nucleus, the pri-miRNA is processed into an ~70 nucleotide long precursor (pre-miRNA), exported into the cytoplasm, and cleaved, resulting in a mature miRNA duplex (21–25 nucleotides long) [58]. After the cleavage, a helicase associated with the RNA-induced silencing complex (RISC) unwinds the miRNA duplex into the guide strand, complementary to the target, and the passenger strand, which is degraded. Using the miRNA as a guide, the RISC complex mediates the binding of the miRNA to the target mRNA, which results in gene silencing; a fully complementary pairing between the miRNA and the mRNA target results, indeed, in the cleavage of mRNA target. On the other side, the presence of some mismatches inhibits the translation by repressing the initial ribosome binding to the mRNA or the ribosome drop off [57,58,59].

Similarly, lncRNAs (about 200 nucleotides long) are transcribed by RNA polymerase II. Generally, they arise from the same locus of a protein-coding gene on the same or opposite strand. Other times their gene sequence falls between two different genes, as a distinct unit [60]. Evidence showed that lncRNAs can regulate the expression on local or distant genes by different mechanisms. lncRNAs can modify the chromatin structure. In particular, they can neutralize the positive charge of the histones, leading to the opening of the chromatin [17], or they can also interact with DNA methyltransferase 1 (DNMT1) preventing or favoring the DNA methylation of their target gene [61,62]. In addition, it has been reported that lncRNAs can also interfere with protein, protein-modifying enzymes and transcription factors thus regulating both the transcription and signaling pathways [63]. Finally, they can also act as miRNA sponge, sequestering and inactivating miRNAs before their binding to the mRNA target [56].

All this increasing evidence suggest that non-coding RNAs have an active role in regulating the gene expression, and, in particular, it has been shown that miRNAs are able to control many biological functions, including DA neuronal development, survival, and death [17]. On the other side, lncRNAs has recently emerged as regulators of gene expression in neuronal development: many of them are involved, indeed, in neurogenesis and cellular differentiation [56].

### 6.2. miRNA-Mediated Control of Differentiation and Maintenance of DA Neurons

miR-125b and miR-181 appear to specifically promote the differentiation of hESCs towards the DA fate, because the percentage of TH^+^ cells is enhanced by the ectopic expression of these two miRNAs [64]. Conversely, in vitro studies on hESCs evidenced that the overexpression of miR-7a led to a decrease of TH^+^ neurons by altering the balance between Wnt and Shh signaling and consequently promoting the switch to glial cell populations; coherently, its knockdown increases the number of DA neurons [65]. Similarly, the hyperexpression of miRNA-124 led to a reduction of TH^+^ neurons.

A miRNA array analysis performed by Yang et al. on purified TH^+^ mESC-derived neurons evidenced that 45 out of 585 miRNAs underwent a more than 5-fold change in expression levels during DA differentiation. Among these, the miR-132 downregulation promoted the differentiation of TH^+^ neurons, whereas its overexpression dampened the yield of DA neurons by downregulating the DA transcription factor Nurr1 [66]. Interestingly, the miR-132 expression can be regulated at transcriptional level: indeed, an independent study highlighted that BDNF regulated miR-132 expression through the ERK-CREB pathway [67].

More recently, another microarray screening identified miR-34b/c among the most upregulated microRNAs during DA differentiation. miR-34b/c is a negative regulator of Wnt signaling and its overexpression increased the yield of TH^+^ cells obtained from mESCs. Moreover, in cells overexpressing miR-34b/c higher levels of *Dat*, *Vmat2*, and *Pitx3* mRNA were observed. Interestingly, the transfection of miR-34b/c, combined with Nurr1 and Achaete–Scute Family BHLH Transcription Factor 1 (Aslc1) doubled the yield of fibroblast trans-differentiation into DA neurons [68].

In addition to their influence on neuronal differentiation, miRNAs can also have roles in mature DA neurons: experiments performed on human DA cells evidenced, indeed, a post-transcriptional regulatory effect of miR-137 and miR-491 on DAT levels: these molecules were able to reduce both genetic and protein expression of DAT binding to its 3′UTR and to influence the dopamine transport; these data suggest that dysregulation of these two miRNAs may affect the DA uptake by regulating DAT expression [69]. miRNAs involved in differentiation and maintenance of DA neurons are summarized in Table 2.

### 6.3. Long Non-Coding RNAs Involved in Development and Function of DA Neurons

Growing evidence supports a key role for lncRNAs in the control of the development and neuronal functions. It has been discovered that the transition from stem cells to neuronal stem cells, progenitors, and fully differentiated neurons is regulated by the interactions between lncRNAs and other factors [56]. The first lncRNA described in DA neurons was rhabdomyosarcoma 2-associated transcript (*Rmst*). In situ hybridization in a mouse model revealed that *Rmst* localized in the midbrain floor plate region, in the IsO and in the anterior neural tube. Successfully, when the DA neuron development region has been established, *Rmst* expression appeared to be restricted to the presumptive DA neurons. However, the putative function of this lncRNA had not deeply investigated [70].

The lncRNA *NONHSAT089477* has been shown to regulate the expression of DA receptors (DRs) DRD3 and DRD5: indeed, the knockdown of this lncRNA led to the downregulation of the expression of these two DRs in a human neuroblastoma cell line. As expected, the overexpression of NONHSAT089477 reverted the phenotype [71]. lncRNAs involved in differentiation and maintenance of DA neurons are summarized in Table 3.

### 6.4. miRNAs Involved in the Pathogenesis of PD

In PD a dysregulation of several non-coding RNAs (e.g., miRNAs and lncRNAs) has been reported [72]. The first study that highlighted the link between miRNAs dysregulation and PD onset was performed by Kim et al. They reported that mice with a deletion of Dicer (the enzyme that cleaves the microRNA precursors into the functional miRNA duplex) had a progressive loss of midbrain DA neurons accompanied by a reduction of mobility, suggesting that Dicer is fundamental for DA neuron differentiation and maintenance [21]. These findings were also supported by Chmielarz that found that PD symptoms in mice became heavier as Dicer decreased [73].

PD is characterized by the selective loss of DA neurons in the substantia nigra and by the accumulation in the affected neurons of Lewy bodies, whose main component consists of α-synuclein (SNCA) [74]. It has been shown that there are miRNAs involved in the regulation of SNCA expression and accumulation. Among them, miR-7a and miR-153 are able to post-transcriptionally regulate the expression of SNCA: miR-7a inhibits its translation, whereas miR-153 degrades its mRNA [75]. In particular, depletion of miR-7a is related with SNCA accumulation and neuron loss in vivo [76]. It has been discovered that PD patients had lower levels of miR-7a in the substantia nigra; in addition, in a mouse model the depletion of miR-7a resulted in a loss of nigral DA neurons and in a reduction of striatal dopamine content [76].

Furthermore, miR-34b/c and miR-214 bind directly SNCA 3′UTR, downregulating its expression [56] and interestingly, miR-34b/c was reported to be reduced in PD brains [77]. In *Drosophila*, the leucine-rich repeat kinase 2 (Lrrk2) carrying the mutation I1915T, which induces a gain of function, antagonized let-7 miRNA, that is a negative regulator of Transcription factor E2f1 (E2f1) and Transcription factor Dp (Dp). It is well known that E2f1 and Dp upregulation in DA post-mitotic neurons may lead to cell death and that they are critical for PD pathogenesis [78]: this suggests that let-7 pathway can be deeply involved in PD pathogenesis. miRNAs can also influence the SNCA accumulation by affecting the FGF20 synthesis; indeed, the rs12720208 polymorphism in the 3′UTR of FGF20 disrupts the binding site for miR-433, increasing the translation of FGF20: this determines a higher SNCA expression both in cell-based system and PD brains [79].

Human LRRK2 is also regulated by miR-205, a miRNA that is expressed at lower level in PD patients with respect to healthy subjects: this observation suggests that the miR-205 downregulation might contribute to the pathogenic increase of LRRK2 protein in patients with sporadic PD [80]. In a cell culture-based model, the administration of miR-205 prevented also the neurite outgrowth defects in neurons carrying the *LRRK2* R1441G mutation [80].

PD patients have a lower level of miR-26a in the cerebrospinal fluid in comparison to healthy subjects and the loss of miR-26a both in a PD mouse model and in PD patients led to an increase of Death-associated protein kinase 1 (DAPK1), that in turn, positively correlates with DA neuron synucleinopathy and death. In particular, the suppression of miR-26a or the upregulation of DAPK1 led to the same phenotype, characterized by DA neuron death, synucleinopathy and motor disabilities in mice [81].

Furthermore, Parkin (PRNK), whose mutation caused a recessive form of early onset PD, is regulated at post-transcriptional level by specific miRNAs. It has been demonstrated that miR-103a-3p downregulates the PRNK expression binding its 3′UTR. In addition, PRNK is a target of other miRNAs, such as miR-181a and miR-218, in particular the latter is reduced in PD patients [75]. On the other side, miR-27a/b can suppress the expression of PTEN Induced Kinase 1 (*PINK1*), a gene involved in the respiratory chain and ATP production. Its mutation is linked with the early onset of PD [75].

Parkinsonism-Associated Deglycase (PARK7) is identified as a recessive familiar PD gene and its downregulation is associated with an early onset of PD due to an increased susceptibility of the cells to the oxidative stress. miR-494 bind PARK7 3′UTR reducing its expression. Very interestingly this miRNA is highly upregulated both in plasma and saliva of PD patients [75].

MiRNAs involved in the pathogenesis of PD are summarized in Table 4.

### 6.5. Long Non-Coding RNA Involved in the Pathogenesis of PD

Furthermore, lncRNAs can play a role in the pathogenesis of different neurodegenerative disease, including PD [56]. Kraus et al. reported that the lncRNA *H19*, *lincRNA-p21*, *Malat1*, *SNHG1*, and *TncRNA* were differentially expressed in PD compared to healthy controls: whereas the first was downregulated, the other lncRNAs appeared to be upregulated [83]. Recently, it has been reported that *Malat1* positively regulated DAPK1 targeting miR-124-3p, contributing to cell apoptosis and motor disorder observed in PD. As expected, *Malat1* knockout reduced DAPK1 expression and decreased the apoptotic rate of DA neurons [83]. Another lncRNA, called *HOTAIR*, has been shown to be involved in PD progression; indeed, it was upregulated in PD mouse model where it improved the stability of *Lrrk2* mRNA, thus promoting the apoptosis of DA neurons [84]. Boros et al. discovered lately that lncRNA *NEAT1* is upregulated in PD patients [85].

The literature also reported lncRNAs playing a protective role in PD, such as *AS-Uchl1*: this molecule induces the expression of Ubiquitin carboxy-terminal hydrolase L1 (*Uchl1*), which play a role in the prevention of cell apoptosis, removing DNA damage [86]. In addition, in a rat model of PD, the downregulation of lncRNA *UCA1* inhibited the activation of PI3K/Akt pathway resulting in a reduction of damage in DA neurons [87]. lncRNAs involved in the pathogenesis of PD are summarized in Table 4.

## 7. Biological and Chemical Approaches to Improve the DA Neurons Survival

### 7.1. Growth Factors and Hormones

Besides the cellular therapy for the replacement of the neurodegenerated cells, another important approach to PD treatment focuses on the attempt to preserve the DA neurons supporting their survival and function. Many efforts have been performed in identifying molecules able to conserve DA neurons and data have been obtained from both transplanted cells and in vitro PD models.

Important evidence has been provided about the role of neurotrophic factors. As reported above, abnormal SNCA folding and aggregation are associated with the PD pathogenesis. Treatment with GDNF efficiently reduced SNA accumulation in DA neurons in vitro [88]. Indeed, the neurotrophic factors such as GDNF activate a signal transduction cascade that support the neurite outgrowth and synaptic plasticity [89]. Although this evidence suggests that neurotrophic factor addition may be a potential method for improving the survival and health of the transplanted cells, clinical trials performed with such biomolecules delivered into the brain tissue either as a protein or gene therapy [90,91] gave inconclusive results [92], thus mitigating the enthusiasm about the possible role of GDNF in the PD treatment.

Ghrelin is a pleiotropic orexigenic hormone that stimulates growth hormone secretion by binding to growth hormone secretagogue receptor 1a (GHS-R1a) [93,94]. Both ghrelin and its receptor are widely present in central nervous system, and DA neurons expression of GHS-R1a results downregulated in a mouse model of PD [95]. Some studies performed on rat models indicate beneficial effects of ghrelin agonists on the non-motor symptoms of PD [96], while in vitro studies indicate that they may antagonize the neurotoxin activity of 1-methyl-4-phenylpyridinium (MPP+) [97].

### 7.2. Chemical Small Molecules

In addition to their role in driving the differentiation process toward the neuronal lineage, some small molecules play a role in supporting the DA neurons survival.

Selberg et al. have characterized small molecules that act as inhibitors of the fat mass and obesity-associated protein (FTO), an RNA N^6^-methyladenosine (m^6^A) demethylase involved in the central nervous system development, neuronal signaling and disease; these compounds evidenced their efficacy in promoting the survival of mouse midbrain DA neurons and rescuing them from growth factor deprivation induced apoptosis [98]. Recently, Renko et al. [99] developed a small molecule named BT44 which mimics the GDNF molecular signal (activation of receptor tyrosine kinase RET and its signaling cascade): BT44 displays protective effects on cultured midbrain DA neurons from the MPP–induced toxicity and promote functional recovery in rats modelling an advanced stage of PD. Being able to penetrate through the blood–brain barrier, such small molecules with neurotrophic activity might represent a promising approach to the PD treatment.

Inhibitors of Histone deacetylases (HDACs) are small molecules that play an important role in modulating the cellular transcription modifying the steady state of chromatin towards hyperacetylation. As in neurodegenerative disease the histone acetylation homeostasis is significantly unbalanced towards hypoacetylation [100] HDACs may represent potential therapeutic targets [101]. HDAC inhibitor phenyl butyrate has evidenced neuroprotective action in a mouse model of PD, reducing the loss of DA neurons. Other HDAC inhibitors (vorinostat and sodium butyrate) were able to rescue cell culture as well as transgenic flies from the toxicity caused by SNCA [102]. However, the observation that TSA (a non-specific inhibitors of all the HDAC isoforms) increases apoptosis of DA neuronal cell lines [103] suggests that the effect of HDAC inhibitors might be multifaceted. Probably, these contrasting results may be ascribable to the non-specificity of TSA that inhibits all the HDACs isoforms (pan-HDAC inhibitor).

The effect of K560, a HDAC 1 and 2 isoform-specific inhibitor, was studied in both in vitro and in animal models: results evidenced its ability to support the DA neurons survival [104]. Similarly, Valproic acid was reported to downregulate the apoptotic caspases (Caspase 3, 7, and 9) and to reduce Bax/Bcl2 ratio in SH-SY5Y cell line treated with the neurotoxin 6-hydroxydopamine (6-OHDA), a widely used molecule for the in vivo and in vitro PD modelling [105].

The 21-aminosteroids (lazaroids) are inhibitors of lipid membrane peroxidation and act as oxygen free radical scavengers. As they exhibit neuroprotective properties, and inhibit some apoptosis pathways, their use in the cell therapy have been tested: interestingly, the treatment of the cells with lazaroids increased the survival of DA neurons during tissue preparation, tissue implantation, and interaction with the host neurons; in addition, it reduced the amount of graft tissue needed for the transplantation [106].

Some studies suggest the hydrogen sulfide (H_2_S) as possible potent therapeutic agent for neurodegenerative diseases. Data obtained in a rat model of PD evidenced that a long-term treatment with NaHS (as donor of H_2_S) attenuates remarkably the sign of Parkinsonism and prevents its progress also increasing the DA neurons survival [107]. Furthermore, Statins (competitive inhibitors of 3-hydroxy-3-methylglutaryl coenzyme A reductase (HMGR) have neuroprotective effects and reduce the loss of DA neuros [108,109] by reducing intracellular SNCA aggregations and restoring neurite degeneration in animal PD models.

### 7.3. miRNAs and lncRNAs Involved in the Survival of DA Neurons and DA Cell Lines

It has been reported that some miRNAs are involved in the survival of DA neurons and their deregulation can contribute to the development of PD [75]. As the loss of DA neurons is responsible for the development of PD symptoms in patients, non-coding RNAs may be consider as a target to prevent the neuronal loss [110].

Kim et al. demonstrated that miR-126 was upregulated in DA neurons localizing in the substantia nigra pars compacta of PD patients. In vitro studies showed that the overexpression of this miRNA increases the vulnerability and consequently the survival of rat primary DA neurons and SH-SY5Y cells after the treatment with the 6-OHDA, downregulating the insulin/IGF1/PI3K/AKT pathway [111].

Furthermore, the miR-200a was upregulated in SH-SY5Y cells after the exposure to MPP+ leading to oxidative stress and cell apoptosis. These data were confirmed by Salimian et al. demonstrating that the miR-200a can induce cellular death via p53 and FOXO signaling pathways. Conversely, the apoptotic rate was reduces inhibiting the miR-200a [112].

On the other side, it has been reported that miRNAs can have a protective role on DA neurons. Both miR-128 and miR-216a protect DA neurons from apoptosis. In particular, miR-216a protects the SH-SY5Y cells from MPP-induced apoptosis downregulating the proapoptotic marker BAX [113].

miR-221 is downregulated in PC12 cells after the treatment with 6-OHDA, but interestingly it has been shown that its overexpression promotes the cell viability, proliferation, and reduced the cell apoptotic rate [75].

Finally, also the expression of miR-326 was found to be downregulated in a PD mouse model. Recent evidence showed that this miRNA plays an important role in the suppression of pyroptotic cell death and thus protects form the development of PD. Indeed miR-326 inhibits many inflammatory factors such as interleukin 1 (IL-1) and 6 (IL-6), Interferon γ (INF-γ) and tumor necrosis factor α (TNF-α). In addition, in a PD mouse model the increase of miR-326 is associated with an increase of DA content and TH expression in the neurons localizing in the substantia nigra pars compacta, also ameliorating the motor dysfunctions [114].

Among the landscape of lncRNAs, it has been demonstrated that NORAD has a role in the maintenance of genome stability, as its downregulation resulted in chromosomal abnormality in human cell lines. In particular. Song et al. reported that NORAD is downregulated in MPP+ treated SH-SY5Y cells, whereas its overexpression reduces the MPP+ cytotoxicity and increases the cell viability [115,116].

miRNAs and lncRNAs involved in the survival of DA neurons and DA cell lines are summarized in Table 5.

### 7.4. Mitochondria Transplantation

As PD is associated with mitochondrial dysfunction, the transplantation of mitochondria isolated from healthy cells has been analyzed. Interestingly, mitochondrial replacement therapy improved the motor symptoms in neurotoxin-induced rat models of PD [117] by restoring the mitochondrial function and the DA neuron health. Recently, also the efficacy of intranasal administration of mitochondria has been tested: this novel nose to brain delivery route appeared less effective than the direct injection in restoring mitochondrial function, while the effects on the neuronal survival and the behavioral improvement were similar [118].

## 8. Engraftment of DA Neurons in In Vivo Models

Different studies investigated the engraftment efficacy and efficiency of hiPSC-derived or hESC-derived DA neurons in PD animal models. Whereas mouse iPSC-derived DA neurons have shown efficacy in PD models [119,120], DA neurons from hiPSCs generally show poor in vivo engraftment [121]. Through the addition of a small molecule that activates canonical WNT signaling, hESC were induced into neural progenitors ranging from telencephalic forebrain to posterior hindbrain fates and transplanted to the striatum of rat PD model, where they engrafted maintaining regional specification and not developing tumors. However, even if the functional symptoms of rats improved, transplanted cells generated DA neurons after transplantation but not forebrain-patterned neurons [42]. Floor plate-based strategies gave rise to human DA neurons that efficiently engraft in vivo, suggesting that past failures might be due to incomplete specification rather than a specific vulnerability of the cells [31]. Kirks et al., in fact, derived engraftable midbrain DA neurons from hiPSCs and demonstrated their in vivo survival and function in three different host species of PD models; in particular, CHIR was used to generate LMX1A+/FOXA2 FP progenitors and, after 25 days of differentiation, NURR^+^ cells were transplanted in immunosuppressed mice, rats and monkeys previously treated with 6-OHDA: transplanted cells showed a robust survival and functionality [35]. Another study used the floor plate induction to generate midbrain DA neurons from hiPSCs: here, CORIN^+^ DA progenitors obtained after 28 of differentiation were grafted into the putamen of a primate model of PD, previously treated with the neurotoxin MPP. Also in this case, transplanted cells did not form tumors in the monkey brains, demonstrate long survival and a functional improvement similar to that obtained with L-DOPA [36].

Summarizing, in vivo studies demonstrated that Foxa2^+^ progenitors are necessary to obtain efficient differentiation of midbrain DA neurons and that modulation of SMAD/SHH signaling and small molecules induction vary transplantation efficiency, cell survival and the motor functional recovery in animal models. Finding the perfect differentiation cocktail and timing may represent a promising therapeutic approach for PD [35,36,42].

## 9. Extracellular Vesicles as Natural Molecules for PD Treatment

Extracellular vesicles (EVs) are particles surrounded by a double layer of phospholipids that are released in response to different stimuli, such as cellular activation, apoptosis induction, inflammation, pathological conditions, and mechanical stress [122]. According to their size, EVs can be classified into three main populations: exosomes (EXOs), microvesicles (MVs) and apoptotic bodies. It has been shown that EVs are involved in cell to cell communication both in short and long distance in vivo [123]. As they can be release in all the body fluid, including plasma, EVs are able to transfer proteins, lipids, miRNAs, and mRNAs over long distances from a donor cell to the target one, altering phenotype of the receiving cell. In addition, thanks to their nanometric size, EXOs released into the plasma can cross the blood–brain barrier, allowing the communication between the central nervous system and the periphery [124]. Recently, it has been shown that the exosomes secreted during the DA differentiation of mouse epiblast-derived stem cells were able to increase the yield of DA neurons obtained from murine ESCs in vitro [22]. On the other side, EVs may be also implicated in some brain disorders, due to their cargo content. Indeed, EXOs can spread pathogenic proteins such SNCA, prions, phosphorylated Tau, and amyloid precursor protein, contributing to the progression of neurodegenerative diseases [125]. In particular, in PD EXOs have been identified as one of the main responsible for the spread of misfolded SNCA from injured neurons to the healthy ones or to glial cells. Nevertheless, the pathway triggering the incorporation of SNCA into EXOs and its release is currently unknown [125]. In 2016, Stuendl et al. demonstrated that the EXOs concentration in cerebrospinal fluid of patients with PD was related with the severity of cognitive impairment [126]. One of the most common causes of inherited PD is an autosomal dominant mutation in *LRRK2* gene, and, interestingly, it has been shown that the mutation R1441C in this gene induces the release of a higher number of EXOs; consequently, Russo et al. speculated that also an increased quantity of toxic SNCA was released in the extracellular space, highly contributing to the spreading of disease to healthy neurons [127].

As EVs can transport biomolecules, natural compounds and drugs across the blood–brain barrier, they represent an attractive therapeutic tool for the treatment of PD. Recently, Qu et al. incorporated the DA into exosomes isolated from mouse blood that where subsequently injected into a mouse model. Data showed that encapsulated dopamine was present in all major organs at higher level than free dopamine, especially in the brain. The PD mouse model was treated for 3 weeks with dopamine-loaded EXOs. Results evidenced a decrease of amphetamine-induced rotation (a common test used to monitor the motor impairment in animal model of PD) in mice treated with dopamine-loaded exosomes in comparison with both saline-treated and unloaded exosome-treated controls [128]. Kojima et al. engineered HEK-293T cells to produce exosomes carrying the mRNA of catalase, known to attenuate neuronal death in PD. Results showed that in Neuro2A cells these exosomes were able to reduce the neurotoxicity of 6-hydroxydopamine (6-OHDA), a drug widely used to mimic the neuronal damage and death typical of PD condition. In addition, they also reduced neuroinflammation in a mouse model of PD [129].

Recently, it has been shown that also EXOs released by human umbilical cord mesenchymal stem cells (hucMSCs) have a neuroprotective role in a PD cellular model. Indeed, Chen et al. demonstrated that EXOs from hucMSCs enhanced the viability and the proliferation rate of SH-SY5Y cells treated with 6-OHDA and reduce the cell apoptosis. These effects were partially reverted using GW4869, an inhibitor of EXOs release. The same group demonstrated that in vivo, hucMSC-derived EXOs can cross the blood–brain barrier reducing the behavioral deficits and the neuronal loss and upregulating the dopamine levels in a PD rat model. Unfortunately, the molecules carried by hucMSCs-derived EXOs were not characterized by the authors [130].

Low doses of Vascular endothelial growth factor (VEGF) have a neuroprotective effect on DA neurons treated with 6-OHDA. A similar outcome was obtained in a rat model of PD [131]. The authors encapsulated VEGF in synthetic vesicles generated using a semipermeable membrane, that were subsequently implanted in the striatum of the PD rats. Data obtained from this study showed that rats receiving the VEGF-containing capsules exhibit a significant reduction of rotational behavior and an increase of TH^+^ fibers, suggesting that VEGF might have an efficacy in the PD treatment [131]. In addition, VEGF seem to promote neuroprotection also in an indirect way, increasing angiogenesis and activating the proliferation of glia [132].

## 10. Conclusions

Preclinical studies have demonstrated the potentiality of cell replacement therapy for NDs, such as PD. However, the availability of an adequate number of cells obtained from an appropriate source and properly differentiated, other than the cell survival rate after transplantation are still a limit that need to be overcome. Investigation about biological molecules that can increase the yield of DA differentiation or that are dysregulated in PD can contribute to develop new therapeutic strategies, to gain the knowledge about the causes related to the onset of NDs, to increase the cell survival after transplantation and consequently the recovery of brain functions. In addition, thanks to the recent development of the RNA medicine, deregulated lncRNAs in PD may be used as therapeutic target for the treatment of this neurodegenerative disease. Indeed, lately antisense oligonucleotides, that are able to regulate the expression of Malat1 and other lncRNAs, have been developed. Even if they have never been tested in PD animal models, they may represent an efficacy therapeutic option for the future. Similarly, the discover that EVs carries molecules that can cross the blood brain barrier reducing the PD symptoms in animal models, suggests that in the future the administration of EVs carrying specific molecules can be administrated to patients for the treatment of PD.

## Figures and Tables

**Table 1:** Schematic overview of some selected dual-SMAD inhibition-based protocols.

Cell Type	Dual-SMAD Inhibition	Molecules Involved in the DA Commitment and Maturation	Sorting	Efficiency	Functional Assay	References
hESCs	SB431542 Noggin	SHH, FGF8, BDNF, AA, GDNF, TGF-β3, cAMP	Not performed	N/A	Not performed	[33]
hESCs	SB431542 LDN193189	SHH, PUR, CHIR99021, FGF8, GDNF, BDNF, cAMP, TGF-β3, DAPT, AA	Not performed	75% TH^+^ cells	Patch clamp, cell transplantation into animal model	[35]
hESCs	SB431542 Noggin	SHH, CHIR99021, GDNF, BDNF, DAPT, cAMP	Not performed	80% FOXA2^+^/LMX1A^+^	Patch clamp, cell transplantation into animal model	[42]
hiPSCs	LDN193189 A8301	FGF8, CHIR99021, GDNF, BDNF, cAMP	CORIN^+^ cells	87% FOXA2^+^/Tubulin beta 3^+^ (TUJI^+^) cells	Patch clamp, cell transplantation in animal model	[36]
hESCs and hiPSCS	SB431542 LDN193189	SHH, PUR, GDNF, BDNF, AA, cAMP, DAPT	CD47^+^ (IAP^+^) cells	N/A	Transplantation into animal model	[37]
hESCs	SB431542 LDN193189	SHH, PUR, CHIR99021	Not performed	86.5% TH^+^ cells	Cell transplantation into animal model	[38]
hiPSCs	SB431542 LDN193189	SHH, PUR, CHIR99021, FGF8, quercetin, GDNF, BDNF, cAMP, TGF-β3, DAPT, AA	Not performed	20% TH^+^ cells	Patch clamp, multi-electrode array (MEA) recording, transplantation into animal model	[40]
Spermatogonial stem cells	SB431542	Retinoic acid, Valproic acid, forskolin, SHH, TGF-β3, FGF8	Not performed	45% TH^+^/TUJI^+^	Calcium imaging, patch clamp, transplantation into animal model	[34]
hFM-MSCs	SB431542 LDN193189	SHH, PUR, CHIR99021, FGF8, GDNF, BDNF, cAMP, TGF-β3, DAPT, AA	Not performed	79% TH^+^ cell	Not performed	[20]

**Table 2:** Schematic overview of some selected protocol for generation of hMOs.

Cell Type	Dual-SMAD Inhibition	Molecules Involved in the DA Commitment and Maturation	Efficiency	Functional Assay	References
hESCs	SB431542 LDN193189	SHH, FGF8, BDNF, AA, GDNF, TGF-β3, cAMP, β-metcaptoethanol, FGF20, Trichostatin A, DAPT	75% TH^+^/FoxA2^+^/NURR1^+^ cells	Not performed	[47]
hiPSCs	SB431542 LDN193189	SHH, FGF8, β-metcaptoethanol, CHIR99021, BDNF GDNF, TGF-β3, cAMP, AA	55% TH^+^ cells	Not performed	[48]
hiPSCs	SB431542 Noggin	SHH, FGF8, β-metcaptoethanol, BDNF, GDNF, cAMP, AA CHIR99021	58% TH^+^ cells	Patch clamp	[49]
hNESCs	Not performed	CHIR99021, SHH, BDNF, AA, GDNF, TGF-β3, cAMP	66.6% TH^+^ cells	Calcium imaging and MEA	[50]
hESCs	Dorsomorphin and A83-01	CHIR99021 gradient, FGF8, SAG, IWP2, BDNF, GDNF, cAMP	85% TH^+^ cells	Patch clamp	[51]

**Table 3:** Schematic overview of miRNAs and lncRNAs involved in the differentiation and maintenance of DA neurons.

Non-Coding RNA	Role	Affected Pathway	References
miRNA-124	Reduction of the percentage of TH^+^ neurons	N/A	[64]
miR-125 miR-181	Enhancing of the differentiation into DA fate, increasing the number of TH^+^ cells	N/A	[64]
miR-132	Reduction of the yield of DA neurons	Downregulation of Nurr1	[67]
miR-34b/c	Reduction of the yield of DA neurons and favors the transdifferentiation of fibroblasts into DA neurons	Negative regulation of Wnt signaling	[68]
miR-137 miR-491	Reduction of DAT expression	Regulation of DA signaling	[69]
Rmst	Midbrain DA neuron specific lncRNA. No data about its function are available	N/A	[70]
NONHSAT089477	Regulation of the expression of DRD3 and DRD5	Regulation of DA signaling	[71]

**Table 4:** Schematic overview of miRNAs and lncRNAs involved in the pathogenesis of PD.

Non-Coding RNA	Role	Observation in PD Patients	References
miR-7 miR-153 miR34b/c miR-214	Downregulation of SNCA expression	miR-7 ↓ miR-34b/c ↓	[56,76,77]
Let-7	Downregulation of E2f1 and Dp	N/A	[78]
miR-205	Regulation of LRRK2 expression	↓	[80]
miR-26a	Upregulation of DAPK1, that correlates with synucleinopathy	↓ in the cerebrospinal fluid	[81]
miR-27a/b	Downregulation of PINK1	N/A	[75]
miR-103a-3p miR-181a miR-218	Downregulation of PRKN	miR-218 ↓	[75]
miR-494	Downregulation of PARK7	↓ in saliva and plasma	[75]
H19 lincRNA-p21, Malat1, SNHG1 TncRN	N/A	H19 ↓ lincRNA-p21 ↑ Malat1 ↑ SNHG1 ↑ TncRN ↑	[82]
Malat1	Upregulation of DAPK1 contributing to the apoptosis of DA neurons	N/A	[83]
Hotair	Increasing of the stability of *Lrrk2*	↑ (in mouse PD model)	[84]
NEAT1	N/A	↑	[85]
AS-Uchl1	Induction of the expression of *Uchl1*, preventing cell apoptosis	N/A	[86]

**Table 5:** Schematic overview of miRNAs and lncRNAs involved in the survival of DA neurons and DA cell lines.

Non-Coding RNA	Role	Observation in PD Patients	References
miR-126	Reducing the survival of DA neurons after treatment with 6-OHDA by insulin/IGF1/PI3K/AKT pathway	↑	[110,111]
miR-200a	Increasing the oxidative stress and cell apoptosis	N/A	[112]
miR-128 miR-216a	Protecting from DA neuron apoptosis	N/A	[113]
miR-221	Promoting cell proliferation, viability and reducing apoptosis	N/A	[75]
miR-326	Protecting from cell death and increasing DA markers in PD mouse models	↓ (PD mouse model)	[114]
NORAD	Contributing to the genome stability and protecting from MPP+ cytotoxicity	N/A	[116]

## Data Availability

Not applicable.
